# Effect of Drying on Heavy Metal Fraction Distribution in Rice Paddy Soil

**DOI:** 10.1371/journal.pone.0097327

**Published:** 2014-05-13

**Authors:** Yanbing Qi, Biao Huang, Jeremy Landon Darilek

**Affiliations:** 1 College of Resources and Environment, Northwest Agriculture and Forestry University, Yangling, Shaanxi, People’s Republic of China; 2 Key Laboratory of Plant Nutrition and the Agri-environment in Northwest China, Ministry of Agriculture, Yangling, Shaanxi, People’s Republic of China; 3 State Key Laboratory of Soil and Sustainable Agriculture, Institute of Soil Science, Chinese Academy of Sciences, Nanjing, Jiangsu, People’s Republic of China; Agroecological Institute, China

## Abstract

An understanding of how redox conditions affect soil heavy metal fractions in rice paddies is important due to its implications for heavy metal mobility and plant uptake. Rice paddy soil samples routinely undergo oxidation prior to heavy metal analysis. Fraction distribution of Cu, Pb, Ni, and Cd from paddy soil with a wide pH range was investigated. Samples were both dried according to standard protocols and also preserved under anaerobic conditions through the sampling and analysis process and heavy metals were then sequentially extracted for the exchangeable and carbonate bound fraction (acid soluble fraction), iron and manganese oxide bound fraction (reducible fraction), organic bound fraction (oxidizable fraction), and residual fraction. Fractions were affected by redox conditions across all pH ranges. Drying decreased reducible fraction of all heavy metals. Cu_residual fraction_, Pb_oxidizable fraction_, Cd_residual fraction_, and Ni_residual fraction_ increased by 25%, 33%, 35%, and >60%, respectively. Pb_residual fraction_, Ni_acid soluble fraction_, and Cd_oxidizable fraction_ decreased 33%, 25%, and 15%, respectively. Drying paddy soil prior to heavy metal analysis overestimated Pb and underestimated Cu, Ni, and Cd. In future studies, samples should be stored after injecting N_2_ gas to maintain the redox potential of soil prior to heavy metal analysis, and investigate the correlation between heavy metal fraction distribution under field conditions and air-dried samples.

## Introduction

Soil heavy metals Cu, Pb, Ni, and Cd, are regarded as “chemical time bombs” because of their propensity for accumulation in the soil and uptake by crops. This ultimately causes human toxicity in both the short- and long-term [1]–[2], making farmland ecosystems dangerous to health [3].

Morphological characteristics and processes of heavy metals have been studied to better understand heavy metal occurrence in various environments, transport pathways, and crop uptake. There are a wide range of soil inorganic and organic substances affected by redox conditions. Redox changes the valence of ions, subsequently affecting the forms of various elements and compounds and their transport processes. Furthermore, heavy metal behavior is strongly correlated with redox potential (Eh) [4].

Many studies of river and lake sediments have reported the effect of redox conditions on the distribution of heavy metals. For example, such changes in redox conditions affect heavy metal association with organic matter (OM) [5], and iron (Fe) and manganese (Mn) oxidized fractions are unstable in reduced environments [3], [6]. Kelderman and Osman [2] reported that in river sediments, exchangeable and carbonate bound fractions of Cu, Zn, and Pb increased as Eh increased, and organic fractions, oxidizable Cu, and oxidizable Pb decreased. Lu et al. [7] found that in reduced Iraqi River sediment in Changchun City, Mn oxide fractions increased significantly, organic bound Cu decreased by 40%, but decrease in organic bound Pb, Zn, and Ni was less than Cu.

Soil heavy metal studies which include plant available indices, total concentrations, and fraction distributions have also been conducted on many agricultural systems, including rice (*Oryza sativa*) production. Rice has a high water requirement compared to other crops and during the growing season, Eh can reach roughly 300–200 mV [4]. Soil samples, including from rice paddies under submerged conditions, are routinely air-dried prior to heavy metal analysis [8]–[12]. Zheng and Zhang [13] studied the effect of moisture regimes on paddy soil heavy metals and found that soil moisture did not affect the direction or pathways of fractionation distribution (from active to stable fractions), but did affect the transformation rate. Zheng and Zhang [13] dried rice paddy soil samples after collection and then reconstituted three moisture regimes under controlled conditions in the lab. It does not appear that sample preparation was conducted in an anaerobic environment, therefore this result does not represent in situ soil moisture regime that controls the distribution of heavy metals.

Redox conditions are known to have a significant effect on heavy metal speciation in sediments [14]. As indicated by Calmano et al. [5], if anoxic sediments are exposed to atmosphere, redox condition change and redistribution and transformation of heavy metal fractions in the sediments takes place. A few studies show the effect of redox conditions on heavy metal availability [15]–[17]. Paddy soil has the anoxic condition during rice growing season similar to the river sediment. Thus, we hypothesize that drying prior to analysis for paddy soil changes the anoxic condition to redox condition as a consequence mobility and uptake of heavy metal by crop are changes. To date, the effect of drying on soil heavy metal fraction distribution in rice paddies is largely unknown.

In order to fill this knowledge gap and work towards a better methodology for sampling and handling paddy soil prior to heavy metal analysis, we conducted a study in Zhangjiagang County, a typical rice production region of China, to 1) understand heavy metal distribution in paddy soil under various redox and pH conditions, and 2) determine the effect of soil air-drying on heavy metal fractions. This study intends to provide useful information for heavy metal assessment in situ. It is hoped that through a better understanding of heavy metal fractions in situ, a better understanding of correlation of heavy metal between soils and crops can be drawn, and a suitable set of corrective measures to prevent heavy metal pollution can be put in place.

## Materials and Methods

### Ethics Statement

All the sample sites were distributed in the private land and permission were approved by the land owner in each site (Zhigang Wang can be contacted for the future permissions). The field studies did not involve endangered or protected species because all the sample sites were in the farmland with rice planted. The coordinates of sample sites ranged from 120°35′ to 120°42′E and from 31°44′ to 31°51′N.

### Sample Collection

Zhangjiagang County was selected as the research area because of two reasons, firstly, the soil is typical paddy soil with rice planting history of more than 100 years, secondly, heavy metals have been accumulated slightly in this area because of sparkly distributed chemical factories. Zhangjiagang County has a humid monsoon climate in the north subtropical zone, with four distinct seasons, and average annual temperature and precipitation is 15.0°C and 1045.9 mm, respectively.

An investigation of soil physical and chemical characteristics of Zhangjiagang County, Jiangsu, was conducted in 2004 [18]–[19]. In the current study, ten locations from the 2004 investigation, each with a long history of rice production, were selected to represent a wide pH range (4.81–8.16).

Topsoil (0–20 cm) was collected in October, 2007 from rice paddies before the harvest and after a full season of submersion. At the time of collection, there was still a 1–2 cm water layer covering the soil. Each soil sample was a composite of sub-samples taken from 6–8 points within 50 m^2^ with a stainless steel soil probe. Half of the sample was quickly placed in polyethylene plastic tubes and the headspace was immediately purged with nitrogen (N) gas [20], pre-treated with acid, and weighed. Once transported to the laboratory, samples were stored under refrigeration at 4°C for the determination of heavy metals fractions under reduction conditions. The remaining sample was placed in polyethylene bags.

### Chemical Analysis

Soil moisture was measured using soil from the polyethylene bags by drying at 105°C for 24 h. The remaining soil from the polyethylene bags were air-dried and sieved through a 2 mm sieve. Soil pH was measured with a glass electrode in a 1∶2.5 soil: water suspension [21]. Soil OM was determined using the dichromate-wet combustion method [22]. The samples were digested using HNO_3_-HClO_4_-HF for measurement of the total heavy metals [19].

The Community Bureau of Reference (BCR) sequential extraction procedure described by Ure et al. [23] was used to extract heavy metal fractions. For the exchangeable and carbonate bound fraction (acid soluble fraction), acetic acid (20 ml, 0.11 mol l^−1^) was added to a 50 ml polypropylene centrifuge tube containing 0.5 g sample and shaken for 16 h at ambient temperature (20±1°C) on an end-over mechanical shaker operating at 40 rpm. The extract was centrifuged at 4000 rpm, decanted into a polyethylene container, and stored at −4°C until analysis. For the Fe and Mn oxide bound fraction (reducible fraction), the residue from the step above was shaken with hydroxylamine hydrochloride (20 ml, 0.1 mol l^−1^, acidified to pH 2 with nitric acid). This was also centrifuged, decanted, and stored as described above. For the organic bound fraction (oxidizable fraction), the residue from reducible fraction was shaken with 30% hydrogen peroxide (20 ml, acidified to pH 2 with nitric acid) and then ammonium acetate (25 ml, 1 mol l^−1^, acidified to pH 2 with nitric acid). This was also centrifuged, decanted, and stored as above. For the residual fraction, the residue from oxidizable fraction was digested using the method described above for the total digestion of heavy metals.

The entire extraction procedure for soil samples in polyethylene tubes was the same as described for the air-dried samples except that samples were handled in an anaerobic chamber, using N as the purge gas. All analysis of heavy metal concentrations was determined using inductively coupled plasma-atomic emission spectrometry (ICP-AES).

### Statistical Analysis

Analysis of variance was performed using the GLM procedure. Mean separations were performed using Duncan’s Multiple Range to test soil pH and redox on soil heavy metal fractions. Linear relationship (correlation) within fractions and regression within the percentage distribution of each metal fraction in oxidized and reduced condition were conducted. All statistical analysis was conducted using SPSS (version 13.0).

## Results

### Soil Physicochemical Properties

Soil physicochemical properties of rice paddies in Zhangjiagang are shown in Table 1. Average soil moisture of submerged soil was 30.2% and ranged from 24.6% to 33.9%. Soil OM ranged from 19.40 g kg^−1^ to 31.30 g kg^−1^ and had a mean of 25.47 g kg^−1^. The most abundant metal was Pb, which had a mean concentration of 43.27 mg kg^−1^. The least abundant metal was Cd, which had a mean concentration of 0.20 mg kg^−1^. The coefficient of variation (CV) for Cd (41.3%) was higher than Cu, Pb, and Ni (25.6%, 8.8% and 9.3%, respectively).

**Table 1:** Soil chemical properties of rice paddies in Zhangjiagang County, Jiangsu.

Samples	pH	Moisture (%)	Soil Organic Matter (g kg^−1^)	Cu	Pb	Ni	Cd
				(mg kg^−1^)			
1	4.81	33.93	31.30	34.87	48.96	28.82	0.236
2	5.54	30.75	26.50	28.35	38.10	26.41	0.087
3	5.87	26.74	26.80	28.57	41.64	24.66	0.160
4	6.12	31.21	28.70	26.64	40.10	25.70	0.190
5	6.45	24.62	23.70	46.71	42.92	30.15	0.107
6	6.94	28.10	29.30	25.73	39.13	24.61	0.135
7	7.17	29.77	19.40	32.56	43.46	31.74	0.180
8	7.61	31.79	21.10	30.99	44.81	28.80	0.327
9	8.02	32.66	26.70	53.48	44.26	28.54	0.300
10	8.16	32.53	21.20	30.26	49.34	24.62	0.281
Average	6.67	30.2	25.47	33.82	43.27	27.41	0.200
St dev	1.10	2.9	3.95	8.66	3.79	2.55	0.083
Cv (%)	16.49	9.7	15.53	25.62	8.76	9.31	41.28

### Soil Heavy Metal Fraction Distribution

**Figure 1:**
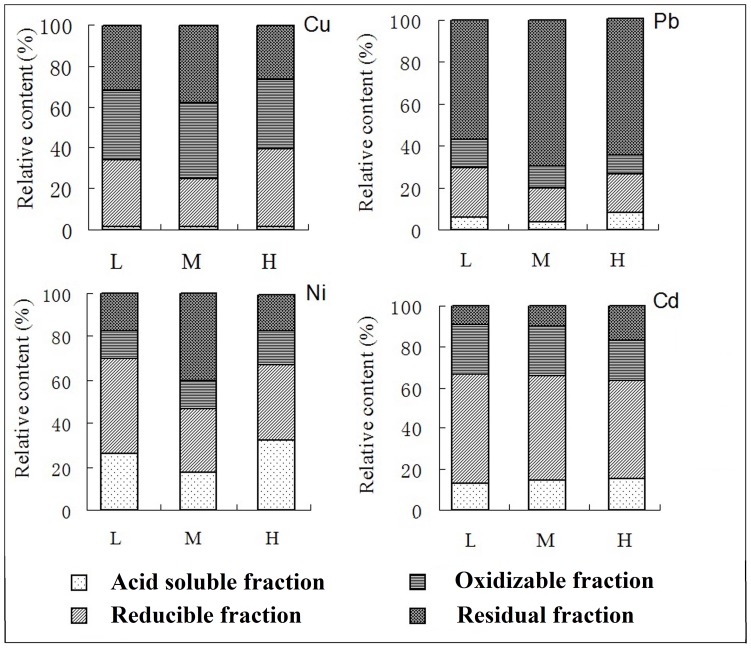
The percentage distribution of each metal fraction in reduced condition in paddy soil samples in Zhangjiagang County. L means lower pH (<6), M means moderate pH (6–7), H means higher pH (>7). Note: significant interactions within and between factors are presented in Table 2.

**Figure 2:**
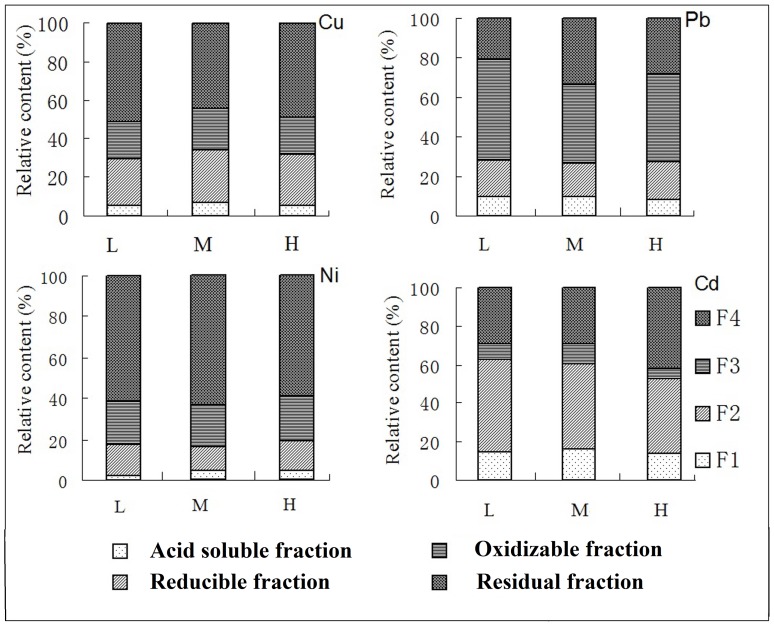
The percentage distribution of each metal fraction in oxidized condition in paddy soil samples in Zhangjiagang County. L means lower pH (<6), M means moderate pH (6–7), H means higher pH (>7). Note: significant interactions within and between factors are presented in Table 2.

Heavy metal fraction distribution varied among elements (Fig. 1 and 2). Redox conditions had a significant effect on all Cu fractions (Fig. 1, Table 2), soil pH did not affect Cu fractions, and there was only a significant interaction effect of redox conditions and pH with reducible fraction. The predominant fractions were reducible fraction and oxidizable fraction (35–40%), followed by residual fraction (15–18%) and acid soluble fraction (<1%). Air-dried samples had significantly different Cu fraction distribution. Compared with reduced soil, acid soluble fraction was still the smallest fraction accounting for 5% of the total Cu. Reducible fraction and oxidizable fraction had significantly lower proportions while residual fraction had significantly higher proportion and was the predominant fraction.

**Table 2:** P-values from the analysis of variance of redox conditions and pH for heavy metal fraction distributions.

Heavy Metal	Factors	P value			
		Acid soluble fraction	Reducible fraction	Oxidizable fraction	Residual fraction
Cu	Redox	<0.001	0.050	<0.001	<0.001
	pH	0.685	0.109	0.325	0.555
	Interaction	0.308	0.034	0.873	0.111
Pb	Redox	0.001	0.901	<0.001	0.000
	pH	0.230	0.544	0.124	0.068
	Interaction	0.123	0.092	0.345	0.970
Ni	Redox	0.000	0.000	0.000	0.000
	pH	0.023	0.009	0.314	0.009
	Interaction	0.040	0.304	0.356	0.164
Cd	Redox	0.08	0.316	0.000	0.024
	pH	0.810	0.261	0.015	0.142
	Interaction	0.089	0.359	0.277	0.413

P-values <0.05 show significant difference within factors and significant interaction between factors for each heavy metal fraction, separately.

Redox conditions affected all Pb fraction distributions except reducible fraction, and pH did not affect fraction distribution (p>0.05) (Table 2). There was also no interaction effect of redox conditions and pH with Pb fractions. In reduced soil, the predominant fraction was residual fraction (60%), followed by reducible fraction (20%), oxidizable fraction and acid soluble fraction. In oxidized soil, acid soluble fraction was 3% higher than in the reduced soil, oxidizable fraction was 20% higher and residual fraction was 33% lower, respectively, than in reduced soil, and reducible fraction was not significantly different.

Redox conditions affected all Ni fraction distributions and pH affected all fraction distributions except oxidizable fraction (Table 2). In reduced soil with pH 6 to 7, Ni_oxidizable fraction_ had the lowest proportion, and Ni_residual fraction_ had the highest (Fig. 1). There was significant interaction of redox conditions and pH with acid soluble fraction. In oxidized soil, acid soluble fraction decreased from 30% to 5% and reducible fraction decreased from 35% to 15%. The residual fraction increased by more than 60% and oxidizable fraction increased less than 5%.

Redox conditions and pH affected Cd_oxidizable fraction_ and Cd_residual fraction_ (Table 2). In reduced soil the predominant fraction was reducible fraction (55%), followed by oxidizable fraction (25%), acid soluble fraction and residual fraction. In oxidized soil, residual fraction increased by 15% and oxidizable fraction decreased by 15%. Treatments had no significant effect on acid soluble fraction or reducible fraction.

### Regression of Reduced and Oxidized Samples

**Figure 3:**
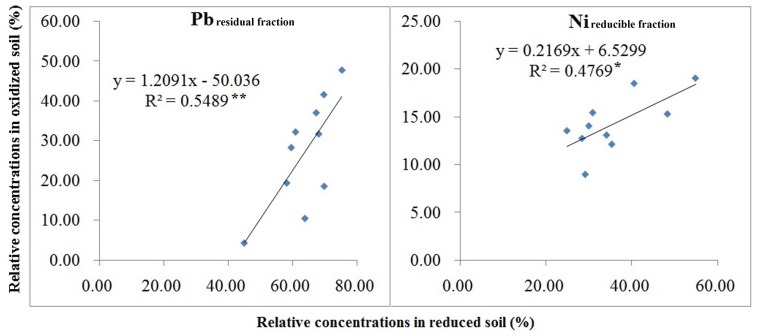
Correlation between the percentage distribution of iron and manganese bound nickel (Ni_reducible fraction_) and residual lead (Pb_residual fraction_) in oxidized and reduced paddy soil samples in Zhangjiagang County. *, and ** denote the significant correlation at *p*≤0.05 and 0.01, respectively.

**Figure 4:**
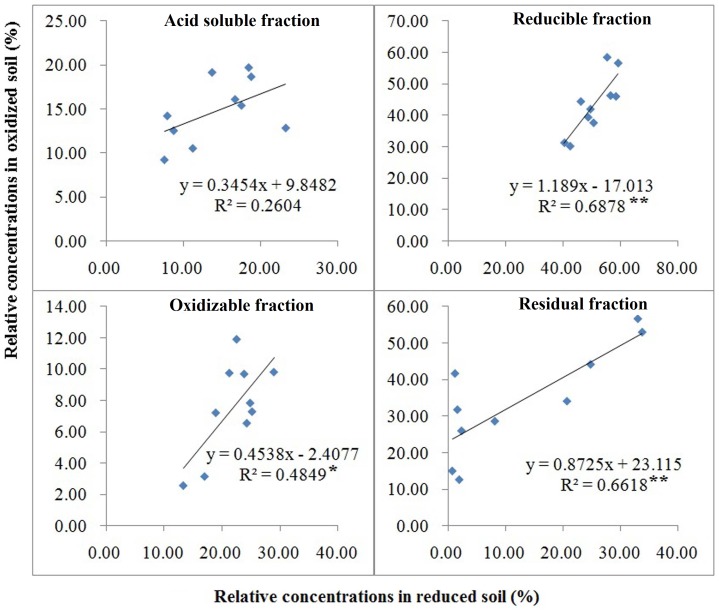
Correlation between the percentage distribution of cadmium in oxidized and reduced paddy soil samples in Zhangjiagang County. *, and ** denote the significant correlation at *p*≤0.05 and 0.01, respectively.

Regression analysis did not reveal significant relationships between soil heavy metal distribution of oxidized and reduced soil. None of the Cu fractions had significant regression results. For Pb, only residual fraction was significant, and for Ni, only reducible fraction was significant (Fig. 3). For Cd fractions, regression of oxidized and reduced samples had *R^2^* values of 0.26, 0.69, 0.48, and 0.66 for acid soluble fraction, reducible fraction, oxidizable fraction, and residual fraction, respectively (Fig. 4). Regression analysis between relative concentration in oxidized soil and relative concentration in reduced heavy soil showed significant correlation for reducible fraction, oxidizable fraction and residual fraction (Fig. 4). Regression analysis separately by pH and OM did not improve correlation between the dependent and independent variable in Fig. 4.

## Discussion

### Total Heavy Metal Concentrations in Rice Paddies

Soil heavy metals originate from both the environment and anthropogenic sources. Although all heavy metal concentrations in this study, except three Cd samples, were higher than typical background concentrations [24], they are lower than the critical limits established by the Soil Environmental Quality Standard of China (GB 15618–1995) [25]. The results of our study are consistent with previous research in the area [25]. In rice paddies of China, there is some evidence that this accumulation is a result of heavy metal rich irrigation water [9]. However, low concentrations of heavy metals suggest that although heavy metal concentrations in Zhangjiagang have experienced accumulation from anthropogenic sources, it is not yet a pressing environmental concern.

### Heavy Metal Fractionation in Reduced Rice Paddies

Generally, rice paddies are submerged during rice cultivation and have lower Eh values during this part of the year. Reduced soil had lower Cu_acid soluble fraction_ and Pb_acid soluble fraction_ and higher Ni_acid soluble fraction_, Ni_reducible fraction_, and Cd_reducible fraction_ than air-dried soil. This is similar to heavy metal fractionation changes that occur in various redox states of sewage sludge [26]–[28]. Förstner and Wittmann [3] explain that for Cu and Pb the most unstable fraction, acid soluble fraction, is replaced by reducible fraction and oxidizable fraction under reduced conditions.

For Ni and Cd, acid soluble fraction accounted for a relatively high proportion (15%–50%), which is not consistent with heavy metal fractionation ranges in sewage sludge. This may be due to Eh differences in rice paddies and sewage sludge, interactions in the rhizosphere of rice paddies, or both. Several studies address changes in soil heavy metal fraction distribution in the rhizosphere [29]–[31]. High Ni_acid soluble fraction_ and Ni_reducible fraction_ indicated that Ni is adsorbed to solid materials or combined with weak acid and Fe and Mn oxides in reduced soil [27]. High Cd_reducible fraction_ was also found in dredged sediments [32].

High Cu_reducible fraction_ and Cu_oxidizable fraction_ in reduced soil indicates that Fe and Mn oxides play a crucial role in Cu processes, and that Fe and Mn oxides cause this fraction to be relatively stable [33]–[34]. This result is consistent with many studies which show dominant oxidizable fraction in reduced sediments [26], [34]–[37].

We found the predominant fraction for Pb in rice paddies to be residual fraction. This fraction is generally considered to be the most stable heavy metal fraction in the soil and unavailable to plants because they are found in the lattice of clay mineral [35], [38]. In addition to residual fraction, sediment Pb in many studies has been found to have large percentages of reducible fraction [2]. Lower reducible fraction in our study might be because of the higher Eh values, interactions in the rhizosphere of rice paddies, or both.

### Effect of Drying on Heavy Metal Fractionation

When soils are routinely dried in the lab prior to chemical analysis, Eh rapidly increases and metals are oxidized. While this may not have a significant effect on heavy metal fractions in agriculture soil which is not submerged, this procedure can drastically change the heavy metal chemistry of reduced soils.

Generally, heavy metal acid soluble fraction increases as Eh increases, however, in our study, Ni and Cd did not respond accordingly. The reason for this is not clear and further study is needed. As Eh increases, reducible fraction generally decreases, most likely because of heavy metal bonding with Fe and Mn oxides [2], [4]–[5], [39]. Our study bears this out as well.

Previous research showed that the relationship of Eh with heavy metal oxidizable fraction and residual fraction is not clear [2]–[5], [7], [39]. In our study, increasing Eh led to increased Pb_oxidizable fraction_ and decreased Cu_oxidizable fraction_. Collavini et al. [40] suggested that decreased Cu_oxidizable fraction_ as soil oxidizes occurs due to the interaction of copper sulfide oxidation and Fe and Mn oxides. Released Cu^2+^ is then redistributed to other fractions such as acid soluble fraction and residual fraction. Part of the obscurity may be because of the complex nature of bonding in copper sulfides, which have both covalent and ionic bonding characteristics and a high occurrence of delocalized electrons [41]–[42].

In this study, increasing Eh led to increased Cu_reducible fraction_, which is in agreement with Kelderman and Osman [2], who reported a decrease in reducible fraction for river sediments under submerged conditions, and with Saeki et al. [14] who reported an increase in Cu_reducible fraction_ after drying of lake sediments.

Drying caused an increase in Pb_oxidizable fraction_, which contradicts previous literature [2], [7]. As Eh increases, Pb_reducible fraction_ slightly decreases, mainly due to bonding of Pb with Fe and Mn oxides [7]. Dried soil had lower Ni_reducible fraction_ and Cd_oxidizable fraction_ and higher Ni_residual fraction_ and Cd_residual fraction_ than reduced soil. This was consistent with the results of other studies [2], [7], [40].

There is strong evidence that drying significantly affects soil heavy metal fractionation of submerged rice paddy soil samples but effects are different than lake sediment, river sediment, and sewage sludge. One of the main reasons most likely is the differences in Eh of the sample sources. Generally, sediment Eh is <−100mv, while rice paddy soil Eh ranged from 200–300 mv [4]. Abundant roots in rice paddies may be another important factor. Root exudates can affect many soil properties such as pH and microbial activity. Changes in these properties can then indirectly affect soil heavy metal fraction distribution. Chen et al. [43] reported that abundant rhizosphere can significantly increase Cu_acid soluble fraction_ and significantly decrease Cd_acid soluble fraction_. Shuman and Wang [44] reported that rice rhizosphere significantly increased Zn_reducible fraction_ and Cu_oxidizable fraction_. Similarly, Lin et al. [24] reported that rice rhizosphere significantly increased Cd_reducible fraction_ and Cd_oxidizable fraction_.

### Effects of Drying on Heavy Metal Availability

The distribution, migration, and plant availability of soil heavy metals are reflected not in the total concentrations, but in specific fractions. With the BCR sequential extraction method, heavy metal bioavailability decreases with each progressively stronger extraction [45]. acid soluble fraction has the highest bioavailability and residual fraction has the lowest. Acid soluble fraction, reducible fraction, and oxidizable fraction are considered active fractions and each contain some bioavailable heavy metals [46]. In this study, Ni and Cd have large active fractions (80%), followed by Cu (>60%), and Pb (<40%). Drying submerged paddy soil samples caused decreases in the active fractions of Cu, Ni, and Cd and increased active fractions of Pb. Zheng and Zhang [13] found an increased bioavailability with increased Eh across all heavy metals measured (Cu, Pb, Cd, and Hg). Our results suggest that when samples are dried prior to heavy metal bioavailability analysis, Pb is overestimated and Cu, Ni, and Cd are underestimated.

Regression analysis results showed that samples which have been dried prior to analysis are not suitable indicators of reduced soil conditions and cannot be used to predict field conditions. However, adding pH and OM did not improve the predictive power, particularly for Cd (data not shown).

### Conclusions

Almost all heavy metal fractions were significantly affected by redox conditions, and few heavy metal fractions were affected by pH. Drying the soil prior to analysis caused metal ions to change valence and a redistribution of heavy metal fractions. Some redistribution occurred as metals moving from active fractions to stable fractions. Therefore, drying decreases the representativeness of *in situ* conditions and the availability of some heavy metals are misestimated. We suggest that soil heavy metal fractionation procedures for rice paddies ensure anaerobic conditions from the time of sampling until analysis. Methodology studies to improve sampling and lab handling techniques would be beneficial, as would further investigation of possible correlation of non-dried sampling and analysis using bioavailable extractions with air-dried samples. For some heavy metals, the processes driving redistribution trends are still unclear. Because the relationship between soil Eh and heavy metal fractionation distribution directly affects heavy metal mobility and bioavailability, a better understanding of the soil heavy metal chemistry under various redox conditions is imperative.
